# Resilience enhancement-oriented traffic police service district division and patrol route planning

**DOI:** 10.1371/journal.pone.0357263

**Published:** 2026-08-28

**Authors:** Yanni Ju, Jinqu Chen, Mengru Ou, Xiaowei Liu, Shaochuan Zhu, Ting Chen, Lijing Chen

**Affiliations:** 1 Department of Road Traffic Management, Sichuan Police College, Luzhou, China; 2 Intelligent Policing Key Laboratory of Sichuan Province, Sichuan Police College, Luzhou, China; 3 Intelligent Policing and National Security Risk Management Laboratory, Sichuan Police College, Luzhou, China; 4 Public Security Department, Fujian Police College, Fuzhou, China; 5 School of Transportation and Logistics, Southwest Jiaotong University, Chengdu, China; 6 Department of Public Security Management, Fujian Police College, Fuzhou, China; Nanjing Forestry University, CHINA

## Abstract

With the increase in car ownership and traffic accidents, optimally designing traffic police service districts and patrol routes (TPSD-PR) has practical significance in improving the capability of an urban road system to cope with traffic events. However, existing studies rarely optimize the TPSD-PR cooperatively with the goal of enhancing urban road system resilience. To address the above-mentioned gaps, a bi-level programming model is developed to optimally design the TPSD-PR in a given region. A solution algorithm combining an improved genetic algorithm and a simulated annealing algorithm is developed to solve the developed model. Case studies performed on a real-world urban road system imply that the formulated model can optimally design its TPSD-PR with the goal of minimizing patrol time and team workload variance. Compared to the current scheme, the total weighted patrol time and the workload variance are decreased by 2.01% and 17.92%, respectively. With the increase in the number of patrol teams, the model’s solution efficiency decreases, while the objective value improves. Historical accident severity directly affects the model’s objective function, implying that the impact of the accident severity on the scheme design cannot be ignored. Finally, the influence of distinct parameters on the model’s effectiveness is discussed. Subsequently, based on the analysis results, several practical suggestions are proposed to improve the resilience of an urban road system under traffic events.

## 1. Introduction

With the further development of urbanization in recent years, the road traffic volume has been increasing day by day, leading to a rise in traffic congestion and accidents, which have seriously affected the urban road traffic safety and system stability [1]. For instance, from 2015 to 2024, the numbers of motor vehicles and traffic accidents in China increased from 279 million to 453 million and 188 thousand to 238 thousand, respectively [2], which have seriously affected the urban road system operation. These incidents act as sudden external shocks that severely degrade the operational performance of the urban road system. Traffic police officers, who are responsible for managing traffic flow and handling accidents, are vital in keeping traffic moving and restoring the normal operation of the affected urban road system. However, in actual operation, the number of traffic police officers is always limited and cannot timely cover every urban road intersection and segment. Hence, it has practical significance to optimally divide traffic police service districts and plan their patrol routes (based on the divided service districts).

Resilience is a comprehensive metric reflecting the ability of a system to cope with external shocks [3,4]. Enhancing the system resilience has practical significance to improve its capability of coping with such shocks. Prior studies usually enhance the urban road system resilience from the transport organization perspective, whereas those from the perspective of optimizing the daily work of traffic managers (e.g., traffic police officers) are still in their initial stage [5]. The logical relation between the traffic police service districts and patrol routes (TPSD-PR) design and system resilience enhancement lies in both proactive and reactive dimensions. Proactively, scientifically planned patrol routes increase police visibility in high-risk areas, which helps eliminate road hazards early and decrease the probability of traffic events (i.e., enhancing the system’s resistance to shocks) [6,7]. Reactively, reasonable service district division and optimal patrol routes ensure balanced spatial coverage, which significantly reduces incident clearance time and prevents secondary congestion. Therefore, optimally designing TPSD-PR serves as a fundamental strategy to enhance the resilience of the urban road system [8].

TPSD-PR are the foundation for traffic police officers carrying out their daily work. First, the service district division is the prerequisite for planning patrol routes. In practice, early works often divide traffic police service districts based on the administrative divisions [9]. However, traffic events like accidents and congestion are highly uncertain and exhibit extreme spatial heterogeneity, implying that the service districts based on the administrative divisions cannot perform well in actual operation. Therefore, researchers have gradually divided service districts by considering other factors like the historical traffic event data [10]. Patrol is an activity conducted by traffic police officers to prevent the occurrence of various traffic events, which is a typical vehicle routing problem [10]. With the goal of minimizing the negative impact caused by traffic events, researchers usually include the influence of the incident hotspot, urban road network structure, and the patrol timing, when they design the traffic police patrol routes.

Although prior studies have conducted numerous works on TPSD-PR, several shortcomings exist. On the one hand, existing studies rarely cooperatively optimize the police service districts and patrol routes by considering the influence of historical accident data. On the other hand, few researchers have included the concept of resilience in their formulated optimization models [11]. To address the above gaps, a bi-level programming model is formulated herein with the goal of minimizing the total weighted patrol time and workload variance among different service districts. A solution approach based on the non-dominated sorting genetic algorithm II (NSGA-II) and the simulated annealing (SA) algorithm is developed to solve the proposed model. Compared to prior studies, main contributions of this paper are summarized as below. On the one hand, a bi-level programming model employed to collaboratively optimize the TPSD-PR is proposed. On the other hand, the formulated model is validated on a real-world urban road system with promising results.

The rest of this paper is organized as follows. First, prior studies on the police service district division and patrol route planning are reviewed in the next section, with its shortcomings being identified later. Section 3 proposes the resilience enhancement-oriented model for collaboratively optimizing TPSD-PR. Subsequently, the model solution approach combining the NSGA-II and SA algorithm is developed. Case studies on a real-world city are performed to validate the model effectiveness in Section 4. Finally, main conclusions and future works are discussed in the last section.

## 2. Literature review

According to the research topic, the following two streams of studies, i.e., police service district division and patrol route planning, are reviewed in the next two subsections. That is, the subsection 2.1 illustrates the existing studies on the police service district division, while the subsection 2.2 reviews the literature on planning patrol routes based on the divided service districts. Then, current research gaps and main contributions of this study are summarized in subsection 2.3.

### 2.1. Police service district division

Traffic police service district division can be regarded as a specific category of the general police district division. However, prior studies on traffic police service district division are rare. Hence, the current studies on police service district division are mainly reviewed herein. Police service district, which is widely known as the police jurisdiction, represents the geographic area where police officers have legal authority to enforce laws [12]. The critical steps in dividing police service districts include police deployment and district planning, which are similar to those in deciding the service districts of warehouses and hospitals [13]. The existing models in dividing police service districts include P-center, P-median, covering models and their variances [14]. The P-center model, which was firstly proposed by Hakimi et al. [15], is a classical model in dividing service districts. It aims at minimizing the maximum distance from any demand point to its nearest facility with the limited number of facilities. For instance, Vlćek et al. [6] utilized the P-center model to decide police service districts. They found that the P-center model performed worse than other classical models.

Unlike the P-center model that is a minimax model, the P-median model divides the service districts by minimizing the total/average distance from all demand points to their nearest facilities with the limited number of facilities [16]. Compared to the P-center model, the P-median model is widely applied in current studies since it is able to focus on high-frequency police incidents while simultaneously ensuring the efficiency of police operations. For instance, Vlćek et al. [6] employed an improved P-center model to plan the police service districts by considering the impact of contiguity and compactness. Lee et al. [17] formulated an improved P-median model to develop an enhanced delineation of police patrol areas in the City of Plano, Texas. Dewinter et al. [18] optimized the police service district in a given region using an enhanced P-center model.

Given that a demand point is within a specified distance of a facility, the covering model decides the police service districts by maximizing the covered demand points or minimizing the number of setting facilities [19,20]. For instance, Curtin et al. [21] developed a novel method to determine the police patrol areas by using a maximum covering model with the goal of maximizing the covered incidents. Price and Curtin [22] utilized the coverage model to determine the police patrol areas by considering the influence of varying demand and facility type. In general, the covering model is the most popular model in deciding the police service districts because of the following bifold advantages. On the one hand, the covering model can better reflect the impact of patrol zones on the service district division. On the other hand, the covering model is easily extensible, allowing to include the constraints of workload and patrol time windows.

### 2.2. Patrol route planning

The police patrol route planning is a kind of complex routing problem, which is similar to the Chinese postman problem and traveling salesman problem [23,24]. Mathematical models are the most commonly used approach for planning patrol routes in prior studies. In accordance with the number of optimization objectives, existing mathematical models employed to design the patrol routes include the single- and multi-objective models [25].

Early research usually utilizes the single-objective model to optimally design the patrol routes [26,27]. The common objective functions defined in the single-objective model consist of optimal resource allocation [28] and the minimal patrol time [29]. For example, Leigh et al. [30] developed an algorithm to design patrol routes in real-time by targeting high crime areas whilst maximizing the demand coverage. Wu et al. [29] formulated a framework that incorporates two game theory models designed for the allocation of police officers to patrol shifts, with the goal of minimizing the total patrol cost. In general, the single-objective model can only optimize a specific objective. It is unable to achieve the collaborative optimization of multiple objectives. In real-world, the traffic police officer patrol routes relate to several factors like available police officers and potential accidents [26], implying that the impact of above factors on the traffic police patrol route planning cannot be ignored.

To address the above gaps, the multi-objective model is gradually emerging in recent years. The objective functions of the prior patrol route multi-objective model include patrol coverage and fairness [31], resource consumption and respond speed [32]. For instance, Wang et al. [33] proposed an integer programing model to optimize the police patrol routes with the goal of maximizing the coverage and minimizing the dispatch cost. Jiang et al. [34] developed a model to design the patrol routes with the goal of minimizing the response time and employed police officers. Although the multi-objective model can obtain more comprehensive patrol routes than the single-objective model, solving the multi-objective model is highly challenging due to its complex mode structure. The above shortcoming significantly limits the model’s practical implication.

### 2.3. Summary

Although prior studies have conducted numerous works on the police service district division and patrol route planning, several shortcomings exist. First, the traffic service district division research neglects the influence of potential traffic accidents. Second, the resilience-oriented traffic police patrol route planning research is still at its initial stage. However, with the further increase in the number of traffic accidents, enhancing the system’s ability to cope with potential accidents is significant. Third, studies that cooperatively optimize the police service districts and patrol routes remains rare.

To the best of our knowledge, the research on cooperatively optimizing the TPSD-PR with the goal of enhancing the urban road system resilience remains at its initial stage. Hence, to address the abovementioned gaps, a bi-level programming model is developed in this study. A solution approach combining the NSGA-II and SA algorithm is proposed to solve the bi-level model. Case studies conducted on a real-world urban road system is utilized to validate the effectiveness of the developed model and solution algorithm.

## 3. Methodology

### 3.1. Problem statement and notations

#### 3.1.1. Problem statement.

**Fig 1:**
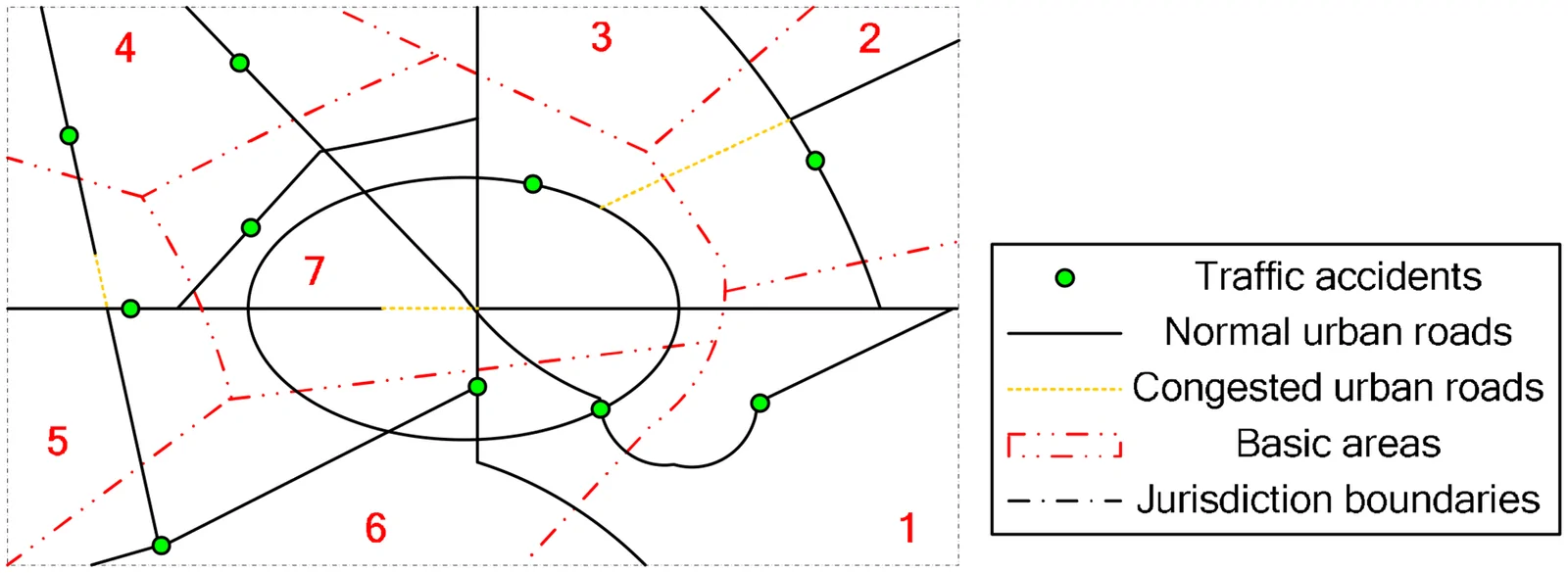
Illustration of problem statement.

In this subsection, a hypothetical rectangular traffic police jurisdiction area shown in Fig 1 is taken as an example to state the studied problem. As shown in Fig 1, seven basic areas (BAs) and three congested urban road segments exist in the studied area. In this study, a BA is defined as an area where traffic accidents frequently occur and the BA is the basic unit for dividing traffic police service districts. In Fig 1, a total of ten historical traffic accidents (shown in the green shaded node) occur in the studied region.

In general, the traffic police department within a given jurisdiction is composed of several subordinate squadrons. The squadrons are responsible for traffic management tasks within their designated areas. Furthermore, some police stations also can handle traffic events. The main research problem of this study is to optimally divide the designated area of each squadron and plan its patrol routes within the designated area, with the goal of improving the system’s ability to deal with potential traffic events and balancing the workload among different squadrons. Assuming that there are two subordinate squadrons in the studied region and two administrative divisions (BAs 1, 5, and 6 belong to the same division, while another division possesses the rest BAs), then the influence of different service district divisions on the emergency response is discussed. When the police service districts are divided based on the administrative division and the squadron centers lie in BAs 1 and 7, respectively, it is found that although the number of accidents in each district is equal, most of congested roads are in the district whose center is BA 7. That is, the workload is imbalanced between two squadrons. Moreover, the patrol routes are directly affected by the location of squadron centers. For the ease of discussion, the squadron is defined as a patrol team (PT) in this study.

The above analysis indicates that the TPSD-PR relates to the urban road network structure, historical traffic events, BAs, and the number of PTs. Moreover, the severity of a given traffic accident relates to several factors like driver behaviors [35,36]. In a city with simple urban road network structure and less traffic events, its TPSD-PR can be directly designed using an enumeration method. However, the above approach does not work well in a real-world city with complex urban road network and high density of traffic events, indicating that a collaborative optimization method used to design TPSD-PR is urgently needed. In this study, a bi-level programming model is developed to optimally design TPSD-PR. Before formulating the model, several assumptions shown below are made:

A1. PTs travel through the shortest path when they patrol between different BAs.

A2. The influence of suddenly occurring traffic accidents on the TPSD-PR scheme design is ignored.

A3. The patrol time at a given BA is positively related to its severity.

#### 3.1.2. Notations.

Main notations involved in this study are listed in Table 1.

**Table 1:** Main notations in this study.

Notations	Descriptions
**Sets**	
K	Set of PTs, k∈K.
N	Set of BAs in the studied region, i,j∈N.
$N_{k}$	Set of BAs assigned to the team k, $i,j\in N_{k}$.
**Parameters**	
$b_{i}$	Workload of the BA i.
$d_{ij}$	Driving time among BAs i and j.
$h_{k}$	Weight of the team k.
$\alpha_{\text{1}}$,$\beta_{\text{1}}$	Model weights in the upper-level model, $\alpha_{\text{1}}+\beta_{\text{1}}=\text{1}$.
$\alpha_{\text{2}}$,$\beta_{\text{2}}$	Model weights in the lower-level model, $\alpha_{\text{2}}+\beta_{\text{2}}=\text{1}$.
$\rho_{i}$	The importance of the BA i.
ξ	Penalty coefficient caused by untimely patrols.
γ	Patrol time required for a unit of accident severity.
$\vartheta_{i}$	Accident severity at the BA i.
χ	Total number of PTs.
**Variables**	
$q_{k}$	$q_{k}\in \left\{\text{0},\text{1}\right\}$, $q_{k}=\text{1}$ implies that team k is assigned a patrol task, and 0 otherwise.
$s_{i}$	$s_{i}\in \left\{\text{0},\text{1}\right\}$, $s_{i}=\text{1}$ means that the BA i is set as the starting point, and 0 otherwise.
$t_{ki}$	$t_{ki}\geq \text{0}$, the arrival time of the team k at the BA i.
$u_{ij}$	$u_{ij}\in \left\{\text{0},\text{1}\right\}$, $u_{ij}=\text{1}$ represents that the BA i is visited before j, and 0 otherwise.
$w_{ij}$	$w_{ij}\in \left\{\text{0},\text{1}\right\}$, $w_{ij}=\text{1}$ denotes that BAs i and j are handled by the same team, and 0 otherwise.
$x_{ki}$	$x_{ki}\in \left\{\text{0},\text{1}\right\}$, $x_{ki}=\text{1}$ implies that the BA i is handled by team k, and 0 otherwise.
$y_{i}$	$y_{i}\geq \text{0}$, the arrival time of a team at the BA i.
$z_{i}$	$z_{i}\in \left\{\text{0},\text{1}\right\}$, $z_{i}=\text{1}$ means that the patrol time at the BA i exceeds the maximum acceptable patrol time, and 0 otherwise.
$\tau_{i}$	$\tau_{i}\in R^{+}$, the patrol time at the BA i.

### 3.2. Model formulation

**Fig 2:**
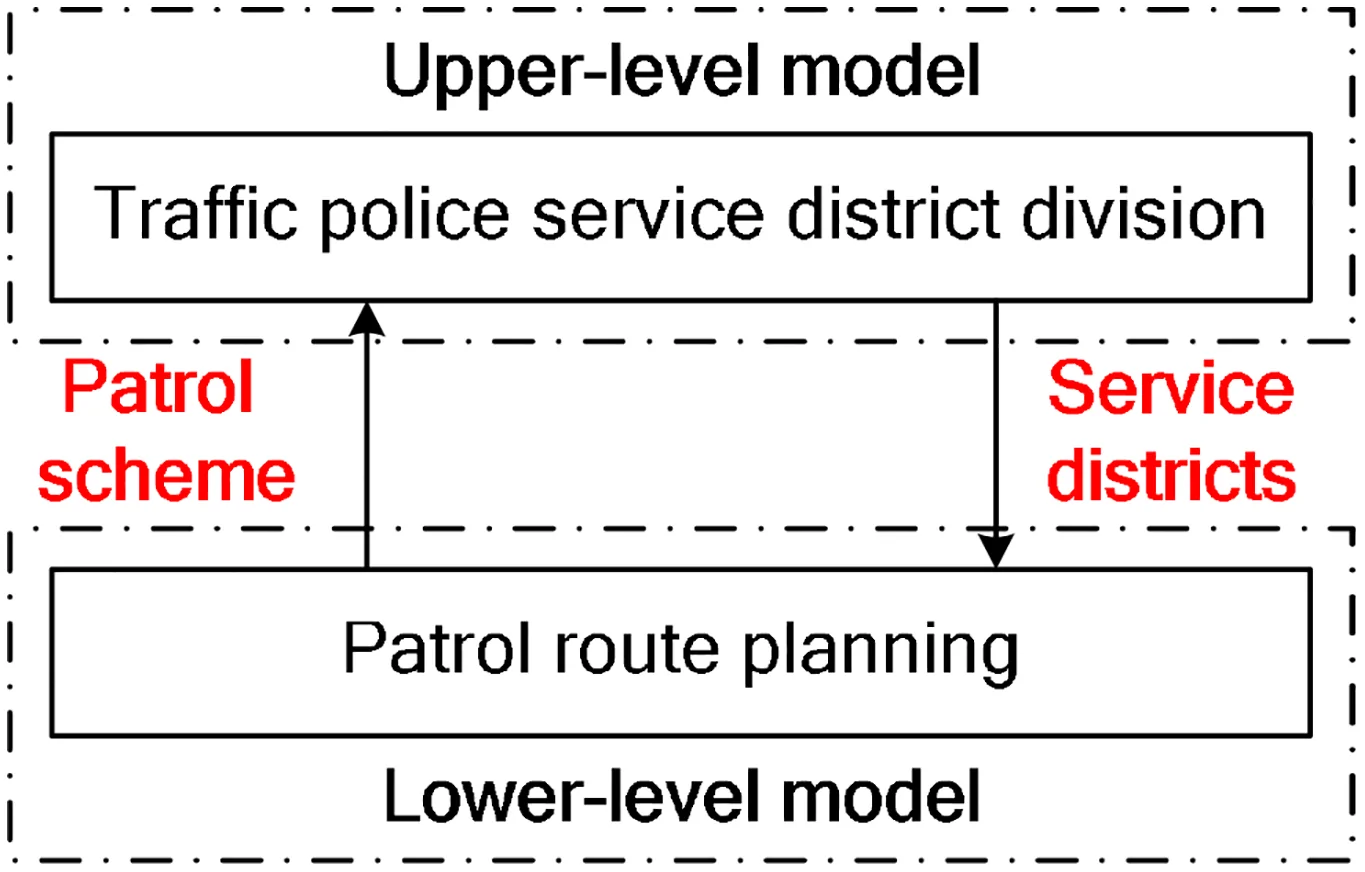
Bi-level programming model framework.

Based on the above discussion, the proposed TPSD-PR strategy enhances the system resilience by optimizing traffic police service districts and patrol routes to reduce the probability and severity of potential traffic accidents, thereby mitigating the impact of traffic accidents on the urban road system operation. Therefore, in this subsection, a bi-level programming model is developed to design TPSD-PR, whose framework is shown in Fig 2. The upper-level model is utilized to divide traffic police service districts, whereas the lower-level model plans patrol routes based on the defined service districts. The upper- and lower-level models interact continuously until the optimal solution is obtained.

#### 3.2.1. Upper-level model.

The upper-level model is utilized to divide traffic police service districts by considering the influence of limited PTs, district contiguity, and compactness, which is presented in Equations (1)–(10) below.


$$\begin{array}{c} \text{min}Z_{\text{1}}=\alpha_{\text{1}}\cdot \sum_{k\in K}\sum_{i\in N}h_{k}\cdot t_{ki}+\beta_{\text{1}}\cdot \xi \cdot \sum_{i\in N}\rho_{i}\cdot z_{i} \end{array}$$ (1)

$$\begin{array}{c} \text{min}Z_{\text{2}}=\text{max}\left(b_{k}\right)-\text{min}\left(b_{k}\right),\;\forall k\in K \end{array}$$ (2)

*s. t.*


$$\begin{array}{c} \sum_{k\in K}x_{ki}=\text{1},\;\forall i\in N \end{array}$$ (3)

$$\begin{array}{c} x_{ki}\leq q_{k},\;\forall i\in N,k\in K \end{array}$$ (4)

$$\begin{array}{c} \sum_{k\in K}q_{k}\leq \chi \end{array}$$ (5)

$$\begin{array}{c} t_{ki}\geq \left(z_{i}-\text{1}\right)\cdot M_{\text{1}}+T+\psi ,\;\forall k\in K,i\in N \end{array}$$ (6)

$$\begin{array}{c} t_{ki}\leq z_{i}\cdot M_{\text{1}}+T,\;\forall k\in K,i\in N \end{array}$$ (7)

$$\begin{array}{c} w_{ij}\geq x_{ki}+x_{kj}-\text{1},\;\forall k\in K,i,j\in N \end{array}$$ (8)

$$\begin{array}{c} w_{ij}\leq \text{1},\;\forall k\in K,i,j\in N \end{array}$$ (9)

$$\begin{array}{c} x_{ki}\in \left\{\text{0},\text{1}\right\},q_{k}\in \left\{\text{0},\text{1}\right\},z_{i}\in \left\{\text{0},\text{1}\right\},t_{ki}\geq \text{0},w_{ij}\in \left\{\text{0},\text{1}\right\} \end{array}$$ (10)

where $\alpha_{\text{1}}$ and $\beta_{\text{1}}$ represent the decision weights, $\alpha_{\text{1}}+\beta_{\text{1}}=\text{1}$. A greater value of the decision weight implies more emphasis on the corresponding sub-objective functions. In this study, $\alpha_{\text{1}}=\beta_{\text{1}}=\text{0}.\text{5}$, implying that the two sub-objective functions are equally important. $b_{k}$ denotes the workload of the team k, which can be measured by the sum of traffic status of BAs belonging to it. $M_{\text{1}}$ is a big constant, which is set as 100,000 in this study. ψ is a small constant, ψ=10 herein.

Equations (1) and (2) define the objective function of the upper-level model, which comprises two sub-objectives. The first sub-objective (displayed in Equation (1)) minimizes the sum of the weighted total patrol time between the squadron center and a given BA and the penalty caused by the failure of team reaching the BA within the maximum acceptable patrol time. The second sub-objective function (shown in Equation (2)) aims to minimize the workload difference among distinct teams. Constraint (3) ensures that a given BA must belong to a team. Constraints (4) and (5) limit the total number of PTs. Constraints (6) and (7) are the linearization results of the constraint shown in Equation (11), which defines the relation between $z_{i}$ and $t_{ki}$. Constraints (8) and (9) imply that BAs belong to the same PT must be contiguous. Constraint (10) defines the domain of the decision variables of the upper-level model.


$$\begin{array}{c} z_{i}=\left\{ \begin{array}{cc} \text{1},\; & t_{ki}>T\\ \text{0},\; & t_{ki}\leq T \end{array}\right. \end{array}$$ (11)

where T is the maximum acceptable patrol time.

#### 3.2.2. Lower-level model.

Based on the service districts determined by the upper-level model, the lower-level model decides the patrol routes within a given district. The lower-level model is a modified traveling salesman problem, which is presented in Equations (12)–(20).


$$\begin{array}{c} \text{min}Z_{\text{3}}=\alpha_{\text{2}}\cdot \sum_{k\in K}\sum_{i\in N_{k}}\sum_{j\in N_{k},\;i\text{≠}j}h_{k}\cdot d_{ij}\cdot u_{ij}+\beta_{\text{2}}\cdot \sum_{k\in K}\sum_{i\in V_{k}}h_{i}\cdot \tau_{i} \end{array}$$ (12)

*s. t.*


$$\begin{array}{c} \sum_{j\in N_{k},j\text{≠}i}u_{ij}=\text{1},\;\forall i\in N_{k} \end{array}$$ (13)

$$\begin{array}{c} \sum_{i\in N_{k},i\text{≠}j}u_{ij}=\text{1},\;\forall j\in N_{k} \end{array}$$ (14)

$$\begin{array}{c} u_{ii}=\text{0},\;\forall i\in N_{k} \end{array}$$ (15)

$$\begin{array}{c} \sum_{i\in N_{k}}s_{i}=\text{1} \end{array}$$ (16)

$$\begin{array}{c} y_{i}\leq M_{\text{2}}\cdot (\text{1}-s_{i}),\;\forall i\in N_{k} \end{array}$$ (17)

$$\begin{array}{c} y_{j}\geq y_{i}+\tau_{i}+d_{ij}-M_{\text{3}}\cdot \left(\text{1}-u_{ij}\right)-M_{\text{3}}\cdot s_{j},\;\forall i,j\in N_{k},i\text{≠}j \end{array}$$ (18)

$$\begin{array}{c} \tau_{i}\geq \gamma \cdot \vartheta_{i},\;\forall i\in N_{k} \end{array}$$ (19)

$$\begin{array}{c} u_{ij}\in \left\{\text{0},\text{1}\right\},s_{i}\in \left\{\text{0},\text{1}\right\},\tau_{i}\in R^{+},y_{i}\geq \text{0} \end{array}$$ (20)

where $M_{\text{2}}$ and $M_{\text{3}}$ are big constants, which are set as 10 and 100,000 in this study, respectively.

As shown in Equation (12), the lower-level model aims to minimize the weighted patrol time in the whole studied region. Constraints (13),(14) guarantee that a given BA can be visited once and the team must go to the next BA once it completes the patrol task, which is used to limit an unbroken patrol routing. Constraint (15) ensures that the team must transition to a distinct BA during its patrol task. Constraint (16) determines that each team must have one starting BA, which is set as its command center herein. Constraint (17) defines the relation between the decision variables $s_{i}$ and $y_{i}$. Constraint (18) limits the arrival time between different BAs. Constraint (19) computes the needed patrol time at a given BA, which is proportional to its severity. Constraint (20) defines the decision variable domains of the lower-level model.

### 3.3. Solution approach

As described in the above subsections, the proposed model is a bi-level programming model. The bi-level programming model is an NP-hard problem, which is hard to be solved by the exact solution algorithm [37]. The NSGA-II can solve the NP-hard problem by efficiently exploring non-linear solution spaces to find high-quality global approximations without requiring mathematical derivatives. Therefore, the formulated model is solved by the NSGA-II herein. Moreover, the NSGA-II is modified by incorporating the SA algorithm to accelerate the convergence. The steps employed to solve the developed bi-level programming model are illustrated in **Algorithm I** below.

**Algorithm I: Bi-level programming model solution approach**

**Input:** Basic data, NSGA-II parameters, and urban road system data.

**Output:** Optimal TPSD-PR.

1. Initialize parameters and populations;

2. Set g=1;// g is the number of generations.

3. **while** $g\;\;\;\;\leq \;\;\;g_{\text{max}}$ **do**// $g_{\text{max}}$ is the maximum number of generations.

4. **for** i **in** P **then**// P denotes the set of population.

5.   Decode the population i to obtain service districts;

6.   Apply SA algorithm to solve the lower-level model;

7.  Compute the fitness value;

8. **end for**

9.  Non-domain sorting and rank the population;

10.  Select the parent population and perform the genetic operations;

11.  Perform the SA algorithm to obtain more optimal offspring;

12.  Non-domain sorting and rank the population;

13.  Record the results;

14.  Update algorithm parameters;

15. **end while**

16. Output results by decoding the optimal population.

As presented in the above pseudocode, the **Algorithm I** begins with initializing parameters and populations based on the input data (i.e., basic data, NSGA-II parameters, and urban road system data). Then, the upper-level model is solved by employing the NSGA-II. When employing the NSGA-II, the chromosome in each population is coded using a real number encoding method. Then, the population is decoded and the SA algorithm is utilized to solve the lower-level model based on the service districts determined by the population. Subsequently, the fitness values of a given chromosome (shown in Equations (1) and (2)) are computed based on the result of the lower-level model. Moreover, to accelerate convergence, the SA algorithm is employed to generate more optimal offspring. Specifically, based on given parameters like the system temperature, the SA algorithm generates offspring through random perturbations and employs the Metropolis criterion to accept inferior solutions with a certain probability. Finally, the optimal traffic police service districts and the patrol routes are outputted by decoding the optimal population.

## 4. Case studies

### 4.1. Description of case studies

#### 4.1.1. Studied urban road system.

To verify the effectiveness of the proposed model, case studies are conducted on a real-world city. Six subdistricts (i.e., Meishan, Ziyun, Huangdun, Sihe, Shuinan, and Shuidong subdistricts) exist in the core of the studied region. It is found that the spatial layout of the urban road network in the studied region is extremely unbalanced. Meishan and Ziyun subdistricts have dense urban road network, while the urban road network density in the rest subdistricts is relatively low. Moreover, it is found that there are many bridges within the studied urban road network, indicating that the failure of critical road segments would have severe impacts on the network operation. Therefore, optimally dividing traffic police service districts and their patrol routes are of significant importance for ensuring the network normal operation and enhancing its resilience.

#### 4.1.2. Data preparation.

**Fig 3:**
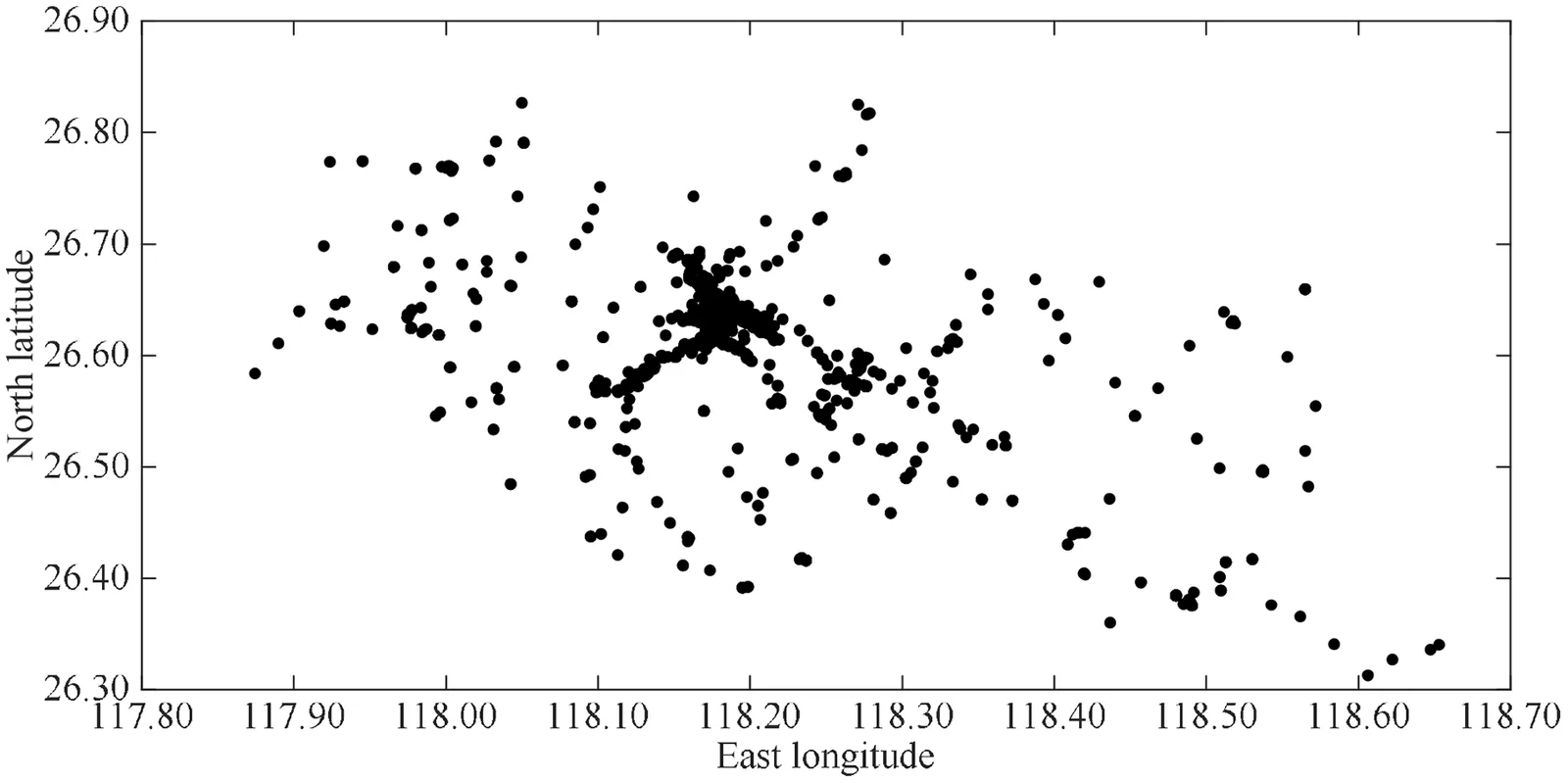
Spatial distribution of traffic accidents.

**Fig 4:**
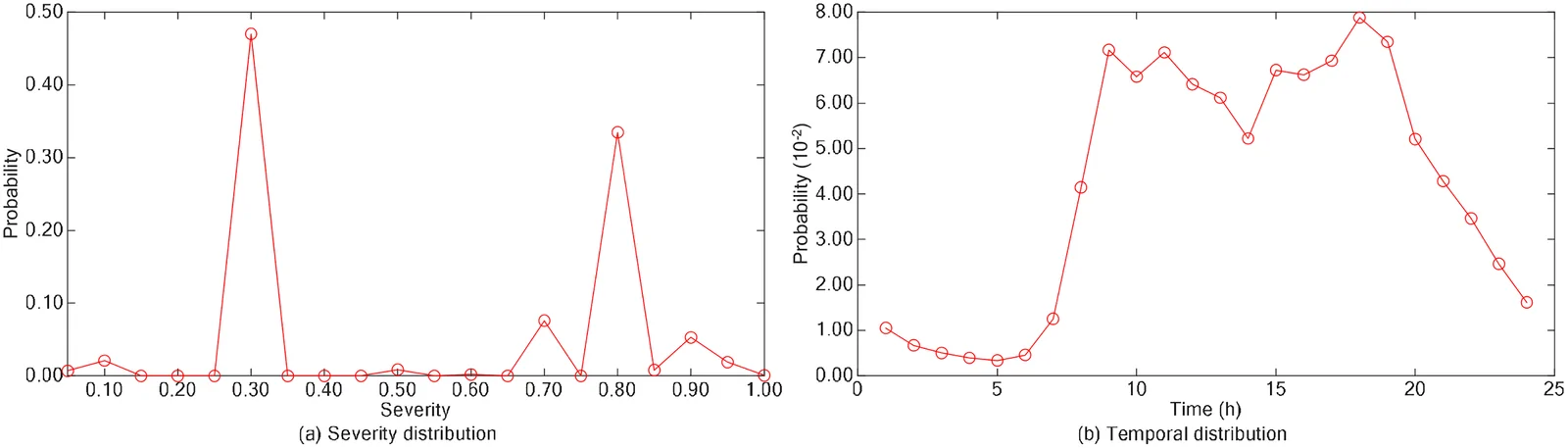
Severity and occurrence time distributions of traffic accidents.

The data on traffic accidents occurring at the studied region in 2024 are obtained from the government managers. The data spatial distribution of the selected data is presented in Fig 3, whose node denotes a traffic accident. Fig 3 indicates that the majority of accidents are located within 26.55°N – 26.70°N and 118.10°E – 118.30°E (accounted for 85.36% of the total amount of data). Hence, this study focuses primarily on traffic accidents occurring within the range of 26.55°N – 26.70°N and 118.10°E – 118.30°E, accidents falling outside this region are excluded. The raw data only provided the accident type, without defining the accident severity [38,39]. Hence, the accident severity is determined with the aid of the most popular large language model from Google (https://gemini.google.com/), which can be found in the following Table 2. In Table 2, a larger value of severity indicates a more severe traffic accident. The severity and occurrence time distributions of the above accidents are shown in Fig 4.

**Table 2:** Severity of different types of traffic accidents.

Accident type	Severity	Accident type	Severity
Accident offense (Criminal)	1.00	Other traffic accidents	0.50
Dangerous driving	0.95	Damage to traffic facilities	0.40
Vehicle-pedestrian collision	0.90	Minor accident (standard procedure)	0.35
Drunk driving	0.85	Minor accident (reported only)	0.30
Vehicle-vehicle collision	0.80	Minor accident (fast-track)	0.30
Single-vehicle accident	0.70	Minor accident (remote assessment)	0.30
Speeding	0.60	Other traffic violations	0.30
Overloading	0.60	Illegal passage	0.15
Unlicensed Driving	0.60	Illegal parking	0.10
Driving scrapped vehicles	0.60	Traffic congestion	0.05

Fig 4(a) presents that the accident severity distribution is highly unbalanced. The accident with a severity of 0.30 (i.e., the minor accident) has the highest occurrence probability. As displayed in Fig 4(b), the probability of occurring traffic accidents during the entire day is greater than that during the night. Moreover, two obvious peaks exist during the entire day, since the traffic volume is great during the morning and evening peak hours. That is, traffic accidents occurring during peak hours have significance impact on the urban road system operation.

**Fig 5:**
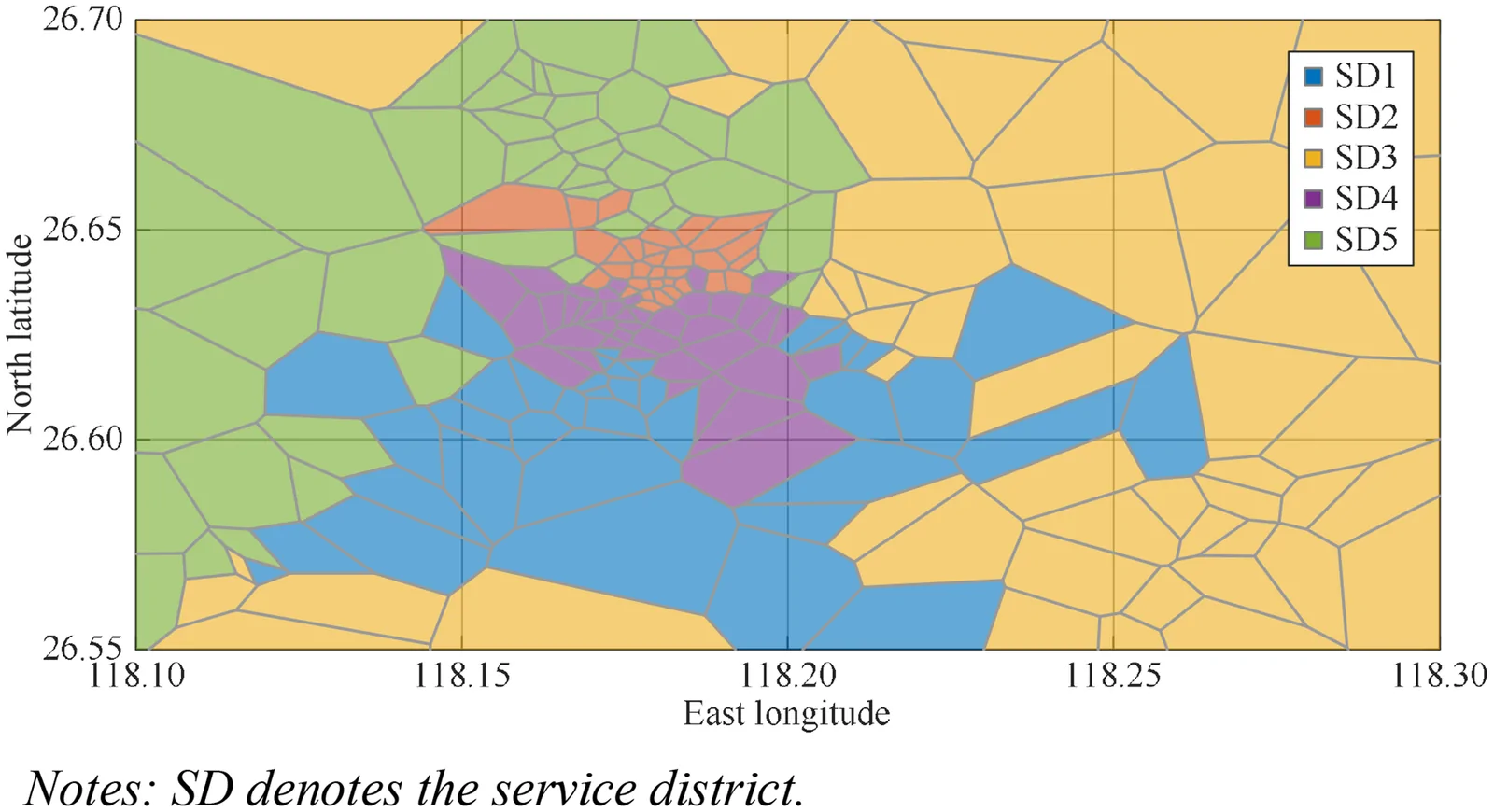
Studied BAs.

The total number of traffic accidents shown in Fig 3 is 11,803, implying that it is hard to directly divide the service districts based on the raw data. Hence, to quickly divide the service districts and decide the patrol routes, the k-means approach is utilized to cluster them into 200 clusters. Based on the Voronoi diagram, the BAs determined by the above clusters are shown in Fig 5. The polygonal region and edge displayed in Fig 5 denote the center and boundary of BAs, respectively. Fig 5 shows that the majority of BAs are located within 26.60°N – 26.65°N and 118.15°E – 118.20°E.

**Fig 6:**
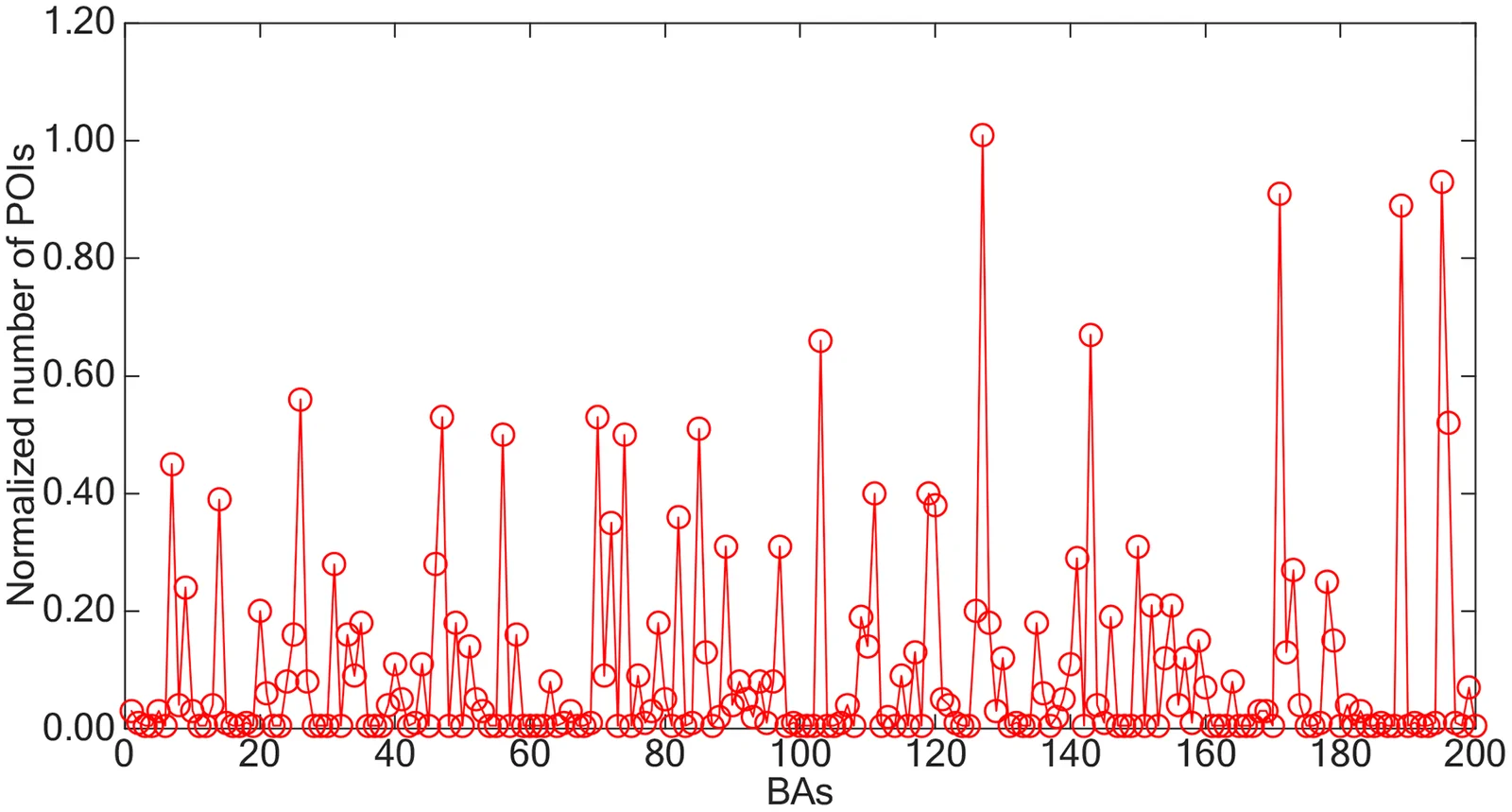
Total number of POI of each BA.

Due to data limitations, the detailed data on traffic status are limited. The Point of Interest (POI) data can reflect the popularity of a given region. Hence, based on the center of each BA, the traffic status of each BA is determined by the POI data. By setting the coverage range as 100 m, the total number of POI of each BA is obtained from the Amap data (https://lbs.amap.com/). For the ease of calculation, the BA whose total number of POI is 0 is set as 0.1 in this study. After normalization, the normalized number of POIs of each BA is presented in Fig 6. The number of POI in the BA whose number is 127 is the highest, while the total number of POI of 79 BAs is 0.1.

The driving time among the above BAs shown in Fig 5 is obtained by processing the Amap data (https://lbs.amap.com/). The maximum and minimal driving time among the above locations are 5,013 s and 44 s, respectively. The average driving time is 1,078.11 s, implying that the driving time among the centers of any two BAs is 1,078.11 s. Finally, other parameters involved in this study are defined in Table 3. The model weights $\alpha_{\text{1}}$, $\beta_{\text{1}}$, $\alpha_{\text{2}}$ and $\beta_{\text{2}}$ are all set as 0.5, indicating that sub-objective functions in objective functions of the upper- and lower-level models are equally important when designing the TPSD-PR. The impact of model weights on designing the TPSD-PR will be discussed later. Due to data limitations, after several tests, the penalty time is set as 1000 s (equaling to 16.67 min). In current scheme, three traffic police squadrons and six police stations exist in the core region. Hence, the total number of PTs is set as 5 in this study. In real world, the time required for traffic police officers to complete a patrol mission depends on the traffic severity. For ease of discussion, the patrol time required for a unit of accident severity is assumed as 60 s. After several tests, the maximum acceptable patrol time for a given BA is set as 18,000 s (i.e., 5 hour).

**Table 3:** Other parameters in this study.

Parameters	Values	Parameters	Values
$\alpha_{\text{1}}$	0.5	ξ	1000 s
$\beta_{\text{1}}$	0.5	χ	5
$\alpha_{\text{2}}$	0.5	γ	60 s
$\beta_{\text{2}}$	0.5	T	18000 s

### 4.2. Result analysis

#### 4.2.1. Optimal schemes.

**Fig 7:**
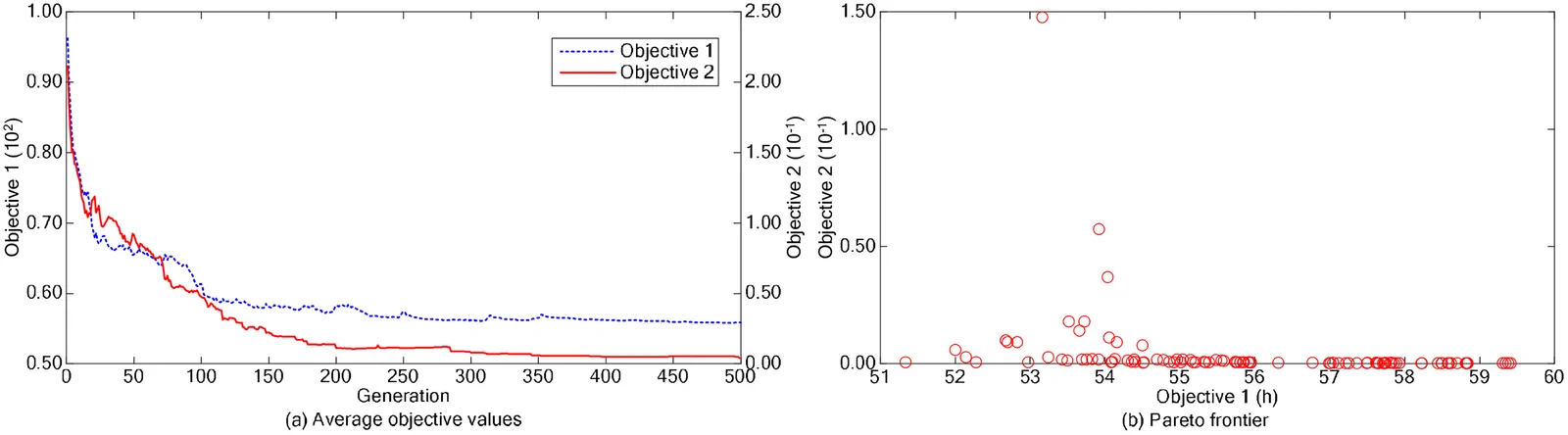
Average objective values and Pareto frontier.

After several tests, the number of generations, the population size, and the cross probability are set as 500, 100, and 0.85, respectively. Then, the proposed bi-level programming model is solved by the formulated solution algorithm. The average objective values during the entire iteration process are calculated and shown in Fig 7(a), indicating that the (near-) optimal solution is obtained. The Pareto frontier determined by the developed algorithm is presented in Fig 7(b).

The solutions determined by the Pareto frontier displayed in Fig 7(b) are all optimal solutions. In actual operation, manager usually aims to minimize the total patrol time. Hence, the Pareto solution with the minimal weighted total patrol time is defined as the global optimal solution in this study. After decoding, the optimal TPSD-PR are presented in Table 4. In Table 4, the “District” and “Patrol routes” columns denote the divided service districts and the optimal patrol routes, respectively. For instance, the bold route belonging to the district D1 represents the patrol route starts and ends at BAs 109 and 1, respectively. Once completing the patrol task, this PT return to BA 109 that is defined as its command center. The total number of BAs in the districts D3 is the highest, while that in the district D2 is the lowest. The BAs belonging to different service districts are presented in Fig 5, implying that the area of the service district in the downtown area is smaller than that in the suburban area. The reason is that the traffic condition in the downtown area is complex and a more refined district helps improve the patrol efficiency. Moreover, the comparison between Figs 3 and 6 reflects that the optimal service district is unrelated to the administrative division. Hence, managers need to include the impact of historical traffic events when designing service districts.

**Table 4:** Optimal schemes.

Districts	Patrol routes
D1	**109-54-68-8-165-62-134-25-181-132-72-22-19-4-180-167-197-195-45-42-460-52-108-66-199-103-124-70-161-96-46-76-79-191-130-27-51-1**
D2	39-120-59-37-127-111-190-32-136-178-140-84-129-88-196-154-13-158-117-3-142-65-200-135-118-133-173-67-26-147-192
D3	6-23-141-64-15-90-172-139-115-36-187-10-188-2-101-107-16-58-24-102-177-33-126-175-41-182-166-61-155-98-143-49-174-184-157-100-92-113-105-69-77-50-171-89-149-9-193
D4	186-94-97-170-152-30-74-47-60-81-119-122-5-128-17-176-93-85-123-34-20-38-75-35-116-125-183-146-162-78-106-144-114-159-21-179-11-110-156-112
D5	43-14-148-145-56-28-44-150-71-73-7-63-189-99-138-151-80-104-194-168-169-82-137-83-163-198-153-18-29-95-57-164-12-40-131-31-121-48-55-91-86-53-185-87

By employing the optimal traffic police service districts and patrol routes shown in Table 4, the objective values of the upper-level model are 51.34 h and $\text{7}.\text{1}\text{1}\times {\text{1}\text{0}}^{-\text{4}}$, respectively, implying that the average total patrol time of a PT is 10.27 h. The maximum and minimum patrol time of the five service districts are 26,228 and 15,816 s, respectively. The variance coefficient among the workload of the above five service districts is 0.16%, implying that the workload is relatively balanced among different districts.

**Fig 8:**
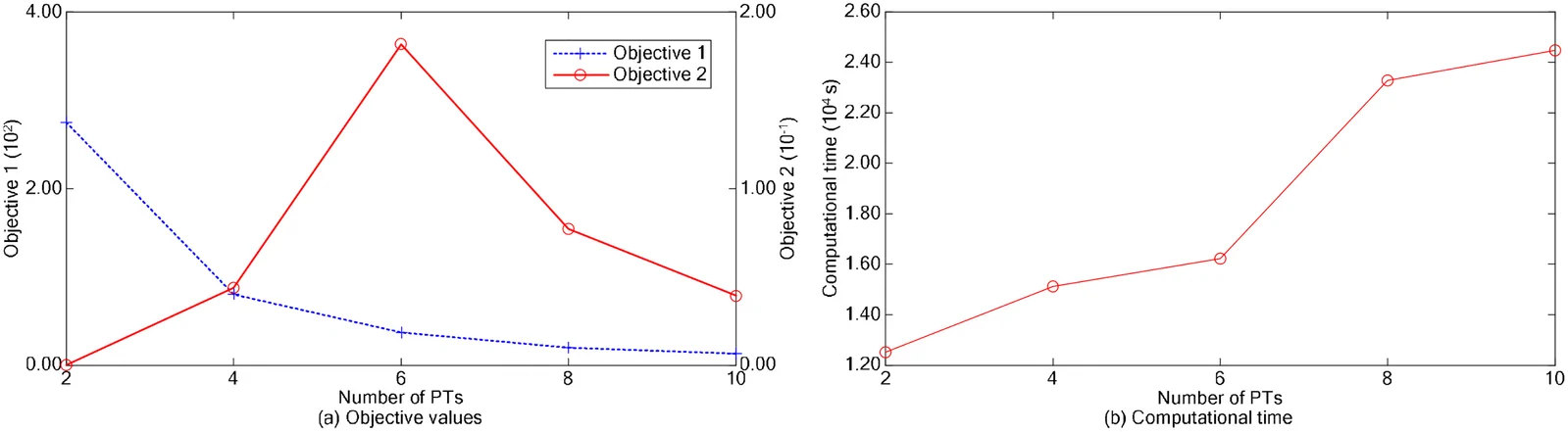
Influence of the number of PTs.

The impact of the number of PTs on the model’s objective values and computational efficiency is presented in Fig 8. Fig 8(a) shows that the value of the objective 1 ($Z_{\text{1}}$) is negatively related to the number of PTs, while there is no obvious relation between the value of the objective 2 ($Z_{\text{2}}$) and the number of PTs. The reason is that the weighted sum of patrol time decreases with the increase in the number of PTs. Fig 8(b) presents that the computational efficiency decreases with the rise of the number of PTs. The above analysis implies that the number of PTs has significant influence on the model effectiveness and computational efficiency. Moreover, increasing the number of PTs in real world requires more investment. That is, the impact of the number of PTs on the TPSD-PR scheme design cannot be ignored.

In this study, the number of clusters is set as 200 (i.e., the total number of studied BAs is 200). To explore the influence of the number of clusters on the model solution, the objective values under distinct number of clusters are computed and listed in Table 5. Table 5 illustrates that the value of objective 1 ($Z_{\text{1}}$) increases with the rise of the number of clusters, while that of objective 2 ($Z_{\text{2}}$) fluctuates with the change of the number of clusters. The reason is that the total patrol time grows with the rise of the number of clusters. Furthermore, it is found that an exponential relation exists between the value of $Z_{\text{1}}$ and the number of clusters, indicating that increasing the number of BAs incurs more workload. Hence, managers should decide the number of studied BAs before they using the formulated model to optimize TPSD-PR.

**Table 5:** Impact of the number of clusters.

NoC	$Z_{1}$ (h)	$Z_{2}$	NoC	$Z_{1}$ (h)	$Z_{2}$
50	2.31	0.33	150	18.84	0.28
100	9.04	0.39	200	51.34	$\text{7}.\text{1}\text{1}\times {\text{1}\text{0}}^{-\text{4}}$

Notes: NoC denotes the number of clusters.

The model’s objective values under different sets of model weights are calculated and tabulated in Table 6. In Table 6, the “Weights” column represents the model weights used in the upper-level model. For instance, the cell “[0.1, 0.9]” represents the values of $\alpha_{\text{1}}$ and $\beta_{\text{1}}$ are 0.1 and 0.9, respectively. Table 6 presents that the value of objective 1 ($Z_{\text{1}}$) is positively related to the model weight $\alpha_{\text{1}}$, while the value of the objective 2 ($Z_{\text{2}}$) is less affected by $\alpha_{\text{1}}$. The reason is that $Z_{\text{1}}$ is positively related to the value of $\alpha_{\text{1}}$, as shown in Equation (1). Moreover, it is found that $Z_{\text{1}}$ is mainly affected by the weighted total patrol time. Hence, to achieve a more resilient TPSD-PR scheme, managers should exactly determine the value of model weights.

**Table 6:** Impact of model weights.

Weights	$Z_{1}$ (h)	$Z_{2}$	Weights	$Z_{1}$ (h)	$Z_{2}$	Weights	$Z_{1}$ (h)	$Z_{2}$
[0.1, 0.9]	14.67	0.04	[0.4, 0.6]	45.48	0.02	[0.7, 0.3]	71.16	0.13
[0.2, 0.8]	25.68	0.12	[0.5, 0.5]	54.15	0.04	[0.8, 0.2]	79.72	0.08
[0.3, 0.7]	35.55	0.01	[0.6, 0.4]	65.59	0.12	[0.9, 0.1]	91.75	0.13

To further validate the effectiveness of the formulated model, the above optimal scheme is compared with the current scheme. In current scheme, three PTs are responsible for patrolling the entire region, with teams 1 and 2 being responsible for the urban area and team 3 being responsible for the rural area. The locations of teams 1–3 are tabulated in Table 7. In the current scheme, the optimal weighted total patrol time and workload variance for the above three PTs are 141.49 h and 0.13, respectively. However, the optimal weighted total patrol time and workload variance decided by the developed model are 138.65 h and 0.11, respectively, when the number of PTs is 3. That is, the weighted total patrol time and workload variance are decreased by 2.01% and 17.92%, respectively, when managers use the formulated model to design the TPSD-PR. The above result indicates that the formulated model helps obtain a more optimal TPSD-PR scheme by minimizing the total weighted patrol time and workload variance.

**Table 7:** Locations of current PTs.

PTs	East longitude	North latitude
Team 1	118.186677	26.637285
Team 2	118.271684	26.591366
Team 3	118.106316	26.573648

**Fig 9:**
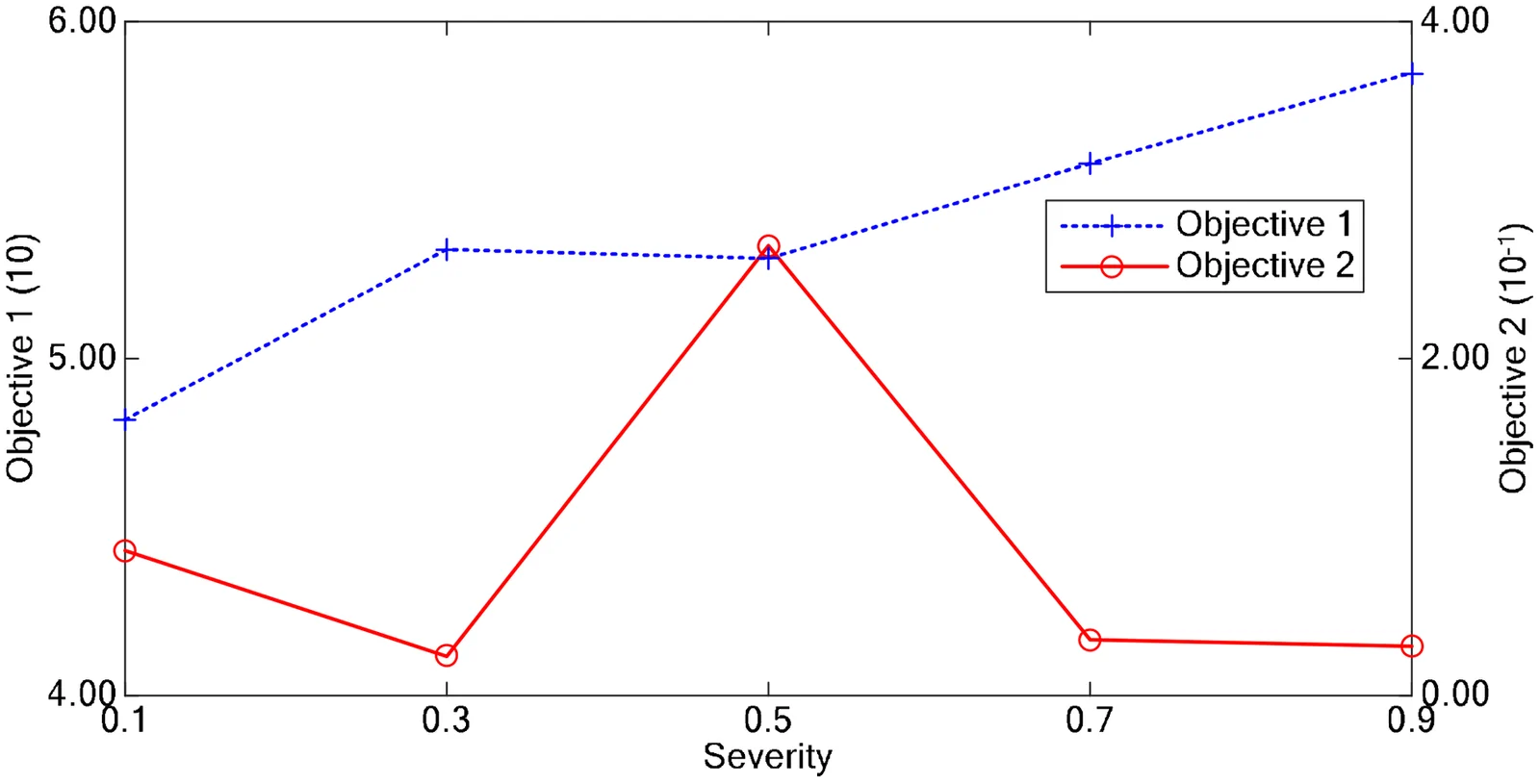
Influence of accident severity.

As described in subsection 4.1.2, the accident severity is determined based on the accident type. To further discuss the influence of the accident severity on the TPSD-PR scheme, the optimal scheme under different accident severity is computed. The corresponding objective values are presented in Fig 9. The *x*-axis in Fig 9 denotes the studied severity. For instance, the severity with a value of 0.1 denotes that the severity of all studied accidents is 0.1. As shown in Fig 9, the value of the objective 1 is positively related to the accident severity (the correlation coefficient between them is 0.958). The reason is that the patrol time at a given BA is affected by its severity. A greater value of the accident severity implies a longer patrol time and a higher value of objective 1 ($Z_{\text{1}}$). However, the value of the objective 2 ($Z_{\text{2}}$) is less susceptible to the accident severity. The above result indicates that the model effectiveness is affected by the accident severity. Managers need to include the influence of the accident severity when they design the TPSD-PR.

#### 4.2.2. Algorithm comparison.

**Fig 10:**
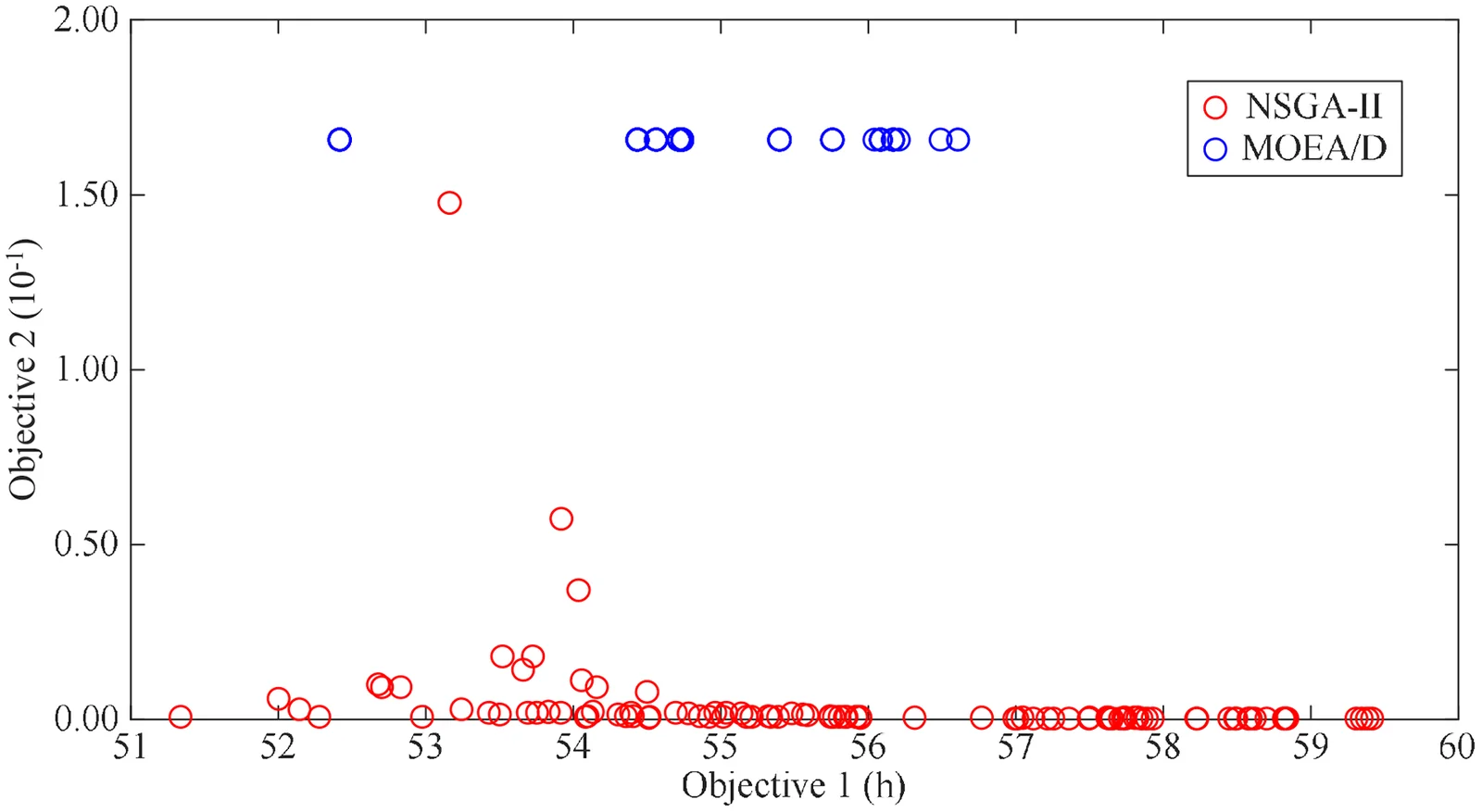
Pareto frontiers determined by different algorithms.

To further reflect the effectiveness of the employed solution approach, the formulated model is solved by the Multi-Objective Evolutionary Algorithm based on Decomposition algorithm (MOEA/D). The Pareto frontiers determined by the MOEA/D and NSGA-II are displayed in Fig 10. The time for applying the MOEA/D and NSGA-II to solve the proposed model is 14,552.55 s and 17,453.24 s, respectively, implying the computational efficiency of the MOEA/D is greater than that of NSGA-II. As displayed in Fig 10, the Pareto frontier determined by the NSGA-II is all located at the lower left of that obtained from the MOEA/D. The optimal objective values obtained by the MOEA/D and NSGA-II are listed in Table 8. The above results imply that the NSGA-II can obtain a more optimal solution than the MOEA/D. To sum up, the developed algorithm combining the NSGA-II and SA algorithm can obtain an optimal solution in a timely manner.

**Table 8:** Optimal objective values determined by different algorithms.

Algorithm	$Z_{1}$ (h)	$Z_{2}$ (10^−4^)
MOEA/D	52.41	1657.58
NSGA-II	51.34	7.11

### 4.3. Sensitivity analysis

**Fig 11:**
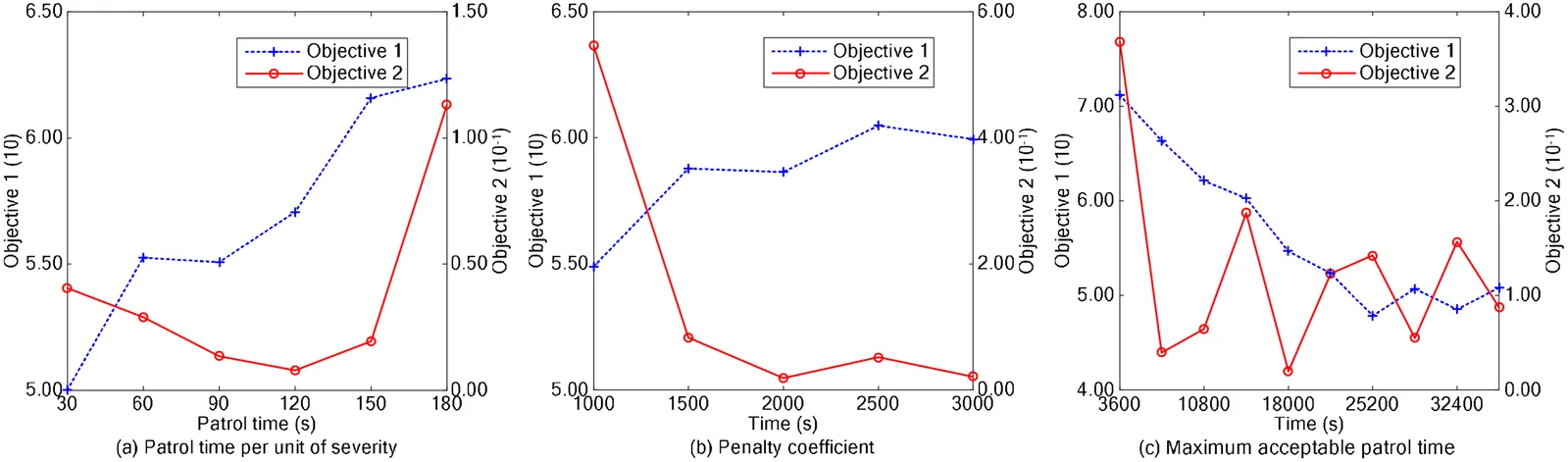
Influence of parameters.

As described in Section 3, the formulated bi-level programming model relates to several critical parameters like the patrol time per unit of accident severity and penalty coefficient. To explore the impact of critical parameters on the TPSD-PR scheme, the controlling variable method is employed in this study. The influence of different parameters on the model is computed and shown in Fig 11.

As shown in Fig 11(a), there is a positive relation between the value of objective 1 ($Z_{\text{1}}$) and the patrol time per unit of severity (the correlation coefficient is 0.964). However, the correlation coefficient between the value of objective 2 ($Z_{\text{2}}$) and the patrol time per unit of severity is 0.451, implying that no obvious relation exists between them. The above result indicates that the patrol time per unit of severity directly affects the weighted total patrol time. In actual operation, a greater value of patrol time at a given BA indicates a smaller probability of occurring traffic accidents. Hence, to obtain an optimal TPSD-PR scheme, managers should determine the value of patrol time per unit of severity by analyzing the historical data. Specifically, traffic police officers can implement a tiered patrol policy. This policy mandates longer and thorough inspection durations for historically high-risk areas to maximize accident prevention, while streamlining procedures in low-risk zones to optimize overall resource efficiency.

Fig 11(b) reflects the relation between the penalty coefficient and objective values, implying that the values of objective 1 ($Z_{\text{1}}$) and 2 ($Z_{\text{2}}$) are positively and negatively related to the penalty coefficient, respectively. The reason is that $Z_{\text{1}}$ is directly affected by the penalty coefficient. A greater value of penalty coefficient implies more severe penalty caused by the failure of PTs reaching the BA within the maximum acceptable patrol time. Although the value of $Z_{\text{2}}$ is not directly affected by the penalty coefficient, a greater value of the penalty coefficient pushes PTs to complete the patrol work quickly while ensuring a balanced workload. That is, the penalty coefficient affects the model effectiveness. Managers should carefully decide the value of penalty coefficient before employing the formulated model. In practice, this result indicates that police agencies should transform this mathematical parameter into strict performance assessment mechanisms. For instance, agencies can use time-based key performance indicator to penalize delayed responses and heavily incentivize rapid, well-balanced patrol executions among frontline teams.

The relation between the objective values and the maximum acceptable patrol time for a given BA is presented in Fig 11(c). Fig 11(c) indicates that the values of objective 1 ($Z_{\text{1}}$) and 2 ($Z_{\text{2}}$) are all negatively related to the maximum acceptable patrol time, since a greater value of the maximum acceptable patrol time means a lower penalty caused by an untimely patrol and more balanced patrol workload. The above result is similar to that displayed in Fig 11(b). In real-world, a reasonable value of the maximum acceptable patrol time helps encourage PTs to design practical patrol routes. Managers can determine the value of the maximum acceptable patrol time by analyzing the historical data or using numerical simulation to determine this value. Therefore, traffic management agencies should utilize this time threshold as a fundamental criterion for deciding the spatial boundaries of traffic police service districts. For instance, for the high-density urban core, enforcing a strict maximum acceptable patrol time will naturally design smaller and highly concentrated districts.

## 5. Conclusions

In an era where traffic events occur increasingly frequently, collaboratively optimizing TPSD-PR scheme has practical significance to improve the ability of an urban road system to cope with potential traffic events. Hence, a bi-level programming model is developed to optimally design TPSD-PR scheme. Case studies conducted on a real-world city is utilized to validate the effectiveness of the formulated model. The main conclusions are summarized below.

First, with the consideration of the influence of historical traffic events, the formulated model can design an optimal TPSD-PR scheme by minimizing the weighted total patrol time and workload variance among different PTs. Second, the model effectiveness is impacted by the accident severity, the number of studied BAs, and the number of PTs. The increase in the number of studied BAs leads to the rise of total patrol time. Compared to the current scheme, the weighted total patrol time and workload variance are decreased by 2.01% and 17.92%, respectively, after employing the optimized scheme. Third, the algorithm comparison between the MOEA/D and NSGA-II reflects that the latter algorithm performs well in solving the model and can obtain a more optimal solution. Finally, the impact of several critical parameters (i.e., patrol time per unit of severity, penalty coefficient, and maximum acceptable patrol time) on the model effectiveness is discussed. It is found that the model effectiveness is severely affected by the above parameters. Managers can determine the above parameters by analyzing the historical data or using numerical simulation.

Although promising results are obtained in this study, several shortcomings exist. First, only the patrol task is considered in this study, without including the influence of sudden traffic events. Second, the patrol time at a given BA is determined by its historical severity herein, without considering the influence of the real-time traffic status. Moreover, the driving time among BAs is assumed to be fixed when solving the formulated model, while the uncertainty in the above driving time is ignored. The above shortcomings will be addressed in future works. A comprehensive model will be developed to obtain a more optimal scheme by considering the impact of sudden traffic events and uncertain driving time. More data on traffic accidents will be employed to further validate the model effectiveness.

## Supporting information

S1 File Supplementary material. https://doi.org/10.1371/journal.pone.0357263.s001.
(RAR)
